# *Rhipicephalus sanguineus* s.l. ticks (Acari: Ixodidae) harbor non-divergent bacterial microbiomes in Arizona

**DOI:** 10.1093/jme/tjaf186

**Published:** 2026-01-22

**Authors:** Maureen Brophy, Kathleen R Walker, Johnathan Adamson, Alison Ravenscraft

**Affiliations:** Department of Entomology & Insect Science, The University of Arizona, Tucson, Arizona 85721, United States; Department of Entomology & Insect Science, The University of Arizona, Tucson, Arizona 85721, United States; Department of Biology, The University of Texas Arlington, Arlington, Texas 76019, United States; Department of Biology, The University of Texas Arlington, Arlington, Texas 76019, United States

## Abstract

*Rhipicephalus sanguineus* (Latreille) is a species complex of ticks that are important vectors of many diseases to humans and other animals. In Arizona, the ranges of the 2 primary genetic variants—the temperate and the tropical lineages—overlap. The temperate and tropical lineages of *R. sanguineus* s.l. have divergent strains of the obligate *Coxiella*-like endosymbiont; however, it is unknown whether the microbiomes of the temperate and tropical lineages are otherwise different. There is growing evidence that non-pathogenic bacteria may be important components of vector-borne disease dynamics, even at low abundance. This research utilized a blocking primer to prevent sequencing of *Coxiella* to enable a closer examination of bacterial community structure of *R. sanguineus* s.l. ticks in Arizona. There were many commonalities among bacterial genera found within *R. sanguineus* s.l. ticks across the state, but no clear distinctions in bacterial community composition based on lineage, sex, female engorgement level, or collection location.

*Keywords:* acarology, insect–symbiont interaction, microbiology, medical entomology

## Introduction

Hematophagous arthropods often have complex relationships with a multitude of bacteria. Ticks are vectors of a broad array of pathogenic microorganisms, transmitting dozens of harmful bacterial agents to humans and other animals (Andreotti et al. 2011, Carpi et al. 2011, Williams-Newkirk et al. 2014, Duron et al. 2015, 2017). In ticks, as in many other blood-feeders, non-pathogenic bacteria play pivotal roles such as supplementing bloodmeals with vitamins and cofactors, or by mediating pathogen infection and transmission (Moran et al. 2008, Guizzo et al. 2017, Duron and Gottlieb 2020). Whether beneficial, pathogenic, or commensal, the members of the tick microbiome may have the capacity to influence their host and, indeed, other bacteria (Macaluso et al. 2002, Bonnet et al. 2017, de la Fuente et al. 2017, Bonnet and Pollet 2020). Therefore, it is critically important to more broadly understand tick bacterial community structure and diversity to gain better insight into their capacity for transmitting diseases to humans and other animals.

While microbiome composition tends to differ across ixodid tick species, several genera of non-pathogenic commensal bacteria are frequent across species, including *Pseudomonas, Sphingobacterium, Acinetobacter, Enterobacter*, and *Stenotrophomonas* (Budachetri et al. 2014, Narasimhan and Fikrig 2015). The overall composition of these microbial communities, however, is highly variable. Ticks encounter and subsequently acquire bacteria throughout their life stages via several routes. Transovarial transmission is a primary means of obligate mutualist and pathogen acquisition, but other bacteria can also take advantage of this method (Bonnet et al. 2017). Ticks may also be exposed to various vertebrate-associated bacteria via their host during feeding, although to what extent this alters the microbiome is still poorly understood, as studies are divided about whether the host’s skin or blood flora influences tick microbiome composition (Hawlena et al. 2013, Rynkiewicz et al. 2015, Zolnik et al. 2016, Chicana et al. 2019, Thapa et al. 2019).

Ticks may also obtain new bacterial passengers through interaction with the environment (Narasimhan et al. 2021). Bacteria commonly associated with soil have frequently been identified in tick microbiome profiles (Zimmer et al. 2013, René-Martellet et al. 2017, Rojas-Jaimes et al. 2021). Hawlena et al. (2013), Chicana et al. (2019), and Thapa et al. (2019) all found that spatial scale and geography can be important in structuring tick bacterial communities. Other factors, including season, temperature, light/dark cycles, host availability, and vegetation, may all influence microbiome composition by imposing stress on the tick host (van Overbeek et al. 2008, Carpi et al. 2011, Lalzar et al. 2012, Williams-Newkirk et al. 2014). Differences between bacterial composition observed in laboratory-reared versus wild-caught ticks highlight the role of environmental constraints as key drivers of bacterial community structure and richness (Narasimhan et al. 2021).

The presence of tick-borne pathogens may also influence bacterial community structure (Steiner et al. 2008, Abraham et al. 2017). Abraham et al. (2017) established that *Anaplasma phagocytophilum* has a large impact on the gut microbiome of *Ixodes scapularis* ticks, partially blocking the development of bacterial biofilm, compromising the integrity of the peritrophic matrix and increasing the tick’s susceptibility to infection. Conversely, pathogen infection can serve as a barrier for infection of other, closely related bacteria (Macaluso et al. 2002, de la Fuente et al. 2003, Gall et al. 2016). Evidence suggests that non-pathogenic bacteria may also directly interfere with pathogen establishment and replication in arthropod species through direct competition for limited resources (nutrients, tissue) or stimulation of the same immune system function (de la Fuente et al. 2003, Bonnet et al. 2017, Bonnet and Pollet 2020). In other cases, non-pathogens may improve conditions for pathogen establishment by suppressing the tick’s immune system (Narasimhan et al. 2014, Bonnet and Pollet 2020).

While summarizing a labile system poses significant challenges, establishing a baseline understanding of microbial richness and composition within ticks can prove an important effort. Zhang et al. (2014) surveyed the microbiome of *Ixodes persulcatus* ticks and their rat host’s blood before and after feeding and found that several bacterial genera identified as members of *Ixodes persulcatus* ticks’ microbiome were also present in the host’s blood after the tick bite, potentially indicating transmission of non-pathogenic bacteria between ticks and mammalian hosts. It is also true that many pathogenic bacteria present in ticks have close non-pathogenic relatives, suggesting a spectrum of intermediate or transitional states between pathogen, commensal, or symbiont (Duron et al. 2015, Bonnet et al. 2017). Examples of this are found in the obligate endosymbiotic bacteria frequently identified across tick species—*Rickettsia, Francisella*, and *Coxiella*—all of which have closely related pathogenic agents (Duron et al. 2015). In fact, it has been observed that some bacterial species could be a mutualist in one host species yet pathogenic in another, leading to a shift in the traditional paradigm of assigning strict “mutualist” or “pathogen” classifications to bacteria (Brownlie and Johnson 2009, Mushegian and Ebert 2016, Zug and Hammerstein 2015). In light of public health preparedness, researchers must be aware of pathogens’ frequent evolutionary shifts and transmission to tick hosts, which offer ample opportunity for the emergence of novel infectious diseases (Bonnet et al. 2017).

The *Rhipicephalus sanguineus* s.l. species complex refers to 11-17 related species or subspecies with a worldwide distribution (Walker et al. 2000, Gray et al. 2013, Dantas-Torres and Otranto 2015). The two dominant lineages, herein referred to as the temperate and tropical lineages, are highly abundant ticks within Arizona. While previously considered sub-taxa of the same species, these closely related but genetically distinct groups exhibit differences in morphology, climactic range limits, and symbiont association, further validating the separation of the 2 lineages into distinct species—*R. sanguineus* sensu stricto (temperate lineage) and *R. linnaei* (tropical lineage) (Dantas-Torres et al. 2013, Nava et al. 2018, Šlapeta et al. 2021, Brophy, Riehle, et al. 2022, Brophy, Walker, et al. 2022). The ranges of the temperate and tropical lineages overlap in at least two counties in Arizona, where latitude and elevation converge to create suitable climatic conditions for both lineages (Brophy, Riehle, et al. 2022).

Recent investigations have compared the microbiome composition of *R. sanguineus* s.l. ticks across broad geographic regions (René-Martellet et al. 2017), within Nigeria (Agwunobi et al. 2021), and between females and embryonic cell cultures of both lineages (de Cassia Luzzi et al. 2021). René-Martellet et al. (2017) found significant differences in the microbiota homogeneity and composition between the temperate and tropical lineages, but this finding is confounded by the continental geographic scale over which samples were collected. Similarly, de Cassia Luzzi (2021) also associated significant differences in bacterial diversity to lineage, though the temperate and tropical ticks were collected approximately 1,000 miles apart in Brazil. The temperate and tropical lineages of *R. sanguineus* s.l. ticks in Arizona harbor divergent strains of *Coxiella*-like endosymbionts (CLEs), with the likely capacity to horizontally acquire the dominant CLE of the opposite lineage in regions of range overlap (Brophy, Walker, et al. 2022).

This study aims to investigate potential similarities and differences in microbial composition and richness among *R. sanguineus* s.l. ticks in Arizona, where both lineages are present and can exist in sympatry with one another. To gain better insight into the *R. sanguineus* s.l. microbiome, we developed a blocking primer to exclude CLEs from amplifying, thus improving the ability to identify patterns in less common bacteria (Vestheim et al. 2011, Gofton et al. 2015). We hypothesized that there would be significant differences in bacterial community structure between the 2 tick lineages and among collection locations.

## Methods

### Sample Collection

Visible *R. sanguineus* s.l. ticks were collected from dogs using fine-tipped forceps and immediately submerged in 70% ethanol in collaboration with local stakeholders during vector control activities, and in animal shelters in communities throughout Arizona. Partner agencies were instructed to collect the least engorged ticks they could find on the dog, but all ticks were accepted. Samples were identified as *Rhipicephalus* using standard taxonomic keys (Walker et al. 2000). Life stage, sex, and engorgement level were assessed as well. Geospatial data are limited to the county to preserve the anonymity of participating collaborators who wish to remain unnamed in print.

### DNA Isolation and Sequencing

Ticks were surface-sterilized with 1-minute successive baths of 8% bleach, sterile PBS buffer, and 100% ethanol, transferred to a 1.5 ml tube with a sterile pestle, submerged in liquid nitrogen to enable homogenization, and treated with 20 mg/ml lysozyme solution. DNA was isolated using the QiaAmp DNA Mini Kit (Qiagen, Hilden, Germany). Lineage of *R. sanguineus* s.l. was determined for all specimens via Sanger sequencing of a 400 bp fragment of the 12S rRNA mitochondrial gene performed at the University of Arizona Genetics Core as described in Brophy, Riehle, et al. (2022). Sequences were manually corrected by visual analysis of the electropherogram, aligned using Geneious Prime 2021.2.2(https://www.geneious.com), and automatically assigned to lineage using R (R Team 2014) based on haplotype nucleotide differences described in René-Martellet et al. (2017) (Accession numbers KU255848-56).

### Blocking Primer

A CLE-specific blocking primer was designed using an alignment of the most common 200 bacterial sequence variants (SVs) from a high-throughput sequencing library created from 99 *R. sanguineus* s.l. ticks from across Arizona using Geneious Prime (Brophy, Walker, et al. 2022). First, the primer design tool was used to identify several candidates that bound to the CLE’s 16S rDNA with favorable annealing temperatures. These candidate primers were then compared with the remaining top 200 common bacterial SVs detected in the ticks, and those predicted to match other bacterial genera were discarded. The top candidate was a 25 bp blocking primer (5′-CAG TGG GGA AGA AAT TCT CAA GGC T-3′) that binds to *Coxiella* rDNA 83 bp downstream of the universal primer binding site. Using Primer BLAST, this blocking primer was compared against the top 5,000 matching NCBI GenBank sequences to ensure it would not bind to any other common bacteria.

### Microbial Sequencing

Bacterial DNA was amplified in a 2-step protocol. First, 16S rDNA was amplified with the 341f/785r primer set described in Klindworth et al. (2013), which amplifies a 464 bp fragment of the V3-V4 hypervariable regions of the 16S rRNA. These primers included an overhang adapter to enable downstream addition of barcodes for sample differentiation. Amplification of CLE 16S rDNA was inhibited with the blocking primer. The PCR protocol used a 20 µl volume per reaction containing 500 nM of each primer, 1 μM CLE blocking primer, and 1× Q5 master mix (New England Biolabs, Ipswich, MA) with 1 μl DNA. Thermocycler conditions were an initial denaturation step at 98 °C for 60 s, 35 cycles of: denaturation at 98 °C for 10 s, blocking primer annealing at 64 °C for 10 s, universal bacterial primer annealing at 58 °C for 20 s, and extension at 72 °C for 30 s, followed by final extension at 72 °C for 120 s. Bacterial amplicons were cleaned with Serapure magnetic bead solution (Rohland and Reich 2012). A second short PCR was used to attach unique pairs of 8-base pair barcodes to the amplicons from each sample (Hamady et al. 2008). The recipe was the same as above, except the CLE blocking primer was omitted and the universal bacterial primers were replaced with indexing primers, assigning a unique pair of forward and reverse barcodes to each sample. Thermocycler conditions were an initial denaturation step at 95 °C for 3 m, followed by 8 cycles of denaturation at 95 °C for 30 s, annealing at 50 °C for 30 s, and extension at 68 °C for 50 s, and final extension at 68 °C for 10 m. Samples were cleaned again with magnetic beads and quantified with Qubit (ThermoFisher, Waltham, MA). We added 12.5 ng of each sample to the final library, which was sequenced on the Illumina MiSeq platform at the University of Texas Arlington. To account for laboratory contaminants, one PCR blank was processed simultaneously with and treated identically to the samples, from the first PCR through sequencing.

### Data Cleaning, Processing, and Statistical Analysis

Illumina primers and indexing primer sites were removed using Cutadapt software (Martin 2011). Inference of bacterial SVs was performed using the DADA2 algorithm, which employs quality scores, the run-specific error rate, and the number of times each sequence was observed to infer the true biological sequences that were present, allowing fine-grained analysis at the level of bacterial strains rather than species (Callahan et al. 2016; R package “dada2” version 1.30.0). Taxonomy of bacterial reads was assigned using the RDP classifier with Silva database version 132 (Wang et al. 2007, Yilmaz et al. 2014). Data management, analysis, and visualization were performed in R version 4.3.3 (R Team 2014) with the “phyloseq” package, version 1.46.0 (McMurdie and Holmes 2013). Mitochondria and chloroplast sequences (which represented 2.1% and 0.9% of the total raw reads, respectively) were removed. Sequences were filtered by read length, retaining reads between 398 and 428 base pairs, inclusive.

To identify laboratory contaminants, we used R’s “decontam” package (version 1.22.0) with the default parameters (Davis et al. 2018) to compare the abundance of each SV in the tick samples versus the PCR blank. Five SVs belonging to the genera *Staphylococcus*, *Methylobacterium-Methylorubrum*, and *Facklamia* were identified as contaminants and removed from the dataset; together, these SVs accounted for 2.7% of the total reads after length filtering. After this, we found that 2 SVs, identified as members of *Staphylococcus* and *Cutibacterium*, were universally present in all samples. The *Staphylococcus* SV accounted for 5% of the PCR blank reads compared to 4% of the total tick reads, and the *Cutibacterium* SV accounted for 2% of reads in the blank versus 4% of the total tick reads. Since presence of a single SV (roughly equivalent to a single bacterial strain) across all samples suggests it may be a laboratory contaminant, we removed these from the dataset. However, low levels of cross contamination often cause common bacteria in samples to appear in sequencing blanks (Davis et al., 2018), and *Staphylococcus* and *Cutibacterium* are both natural inhabitants of dog skin (Tang et al. 2020). Finally, *Coxiella* sequences were removed.

Unless otherwise stated, analyses were performed on data rarefied to 11,197 reads per sample to normalize for differences in sequencing depth among samples. This rarefaction depth was chosen based on visual inspection of rarefaction curves, selecting the lowest depth at which a sample was sequenced and the curves had flattened out.

PERMANOVA (the adonis2 test in the R package “vegan” version 2.6.10; Oksanen et al. 2017) was used to test for dissimilarity in community composition between lineages, sexes, and female engorgement levels. Differential abundance testing was performed on unrarefied data using the “DESeq2” package (version 1.42.1) as adapted for microbiome data (Anders and Huber 2010, McMurdie and Holmes 2013). To assess whether geographic location impacted bacterial community structure, coordinates for each sample were assigned based on nearest city or town to the collection location and we calculated the Bray-Curtis dissimilarity distance between *R. sanguineus* s.l. samples as a function of geographical distance using the “geosphere” package (version 1.5.20) in R.

## Results

**Fig. 1.**
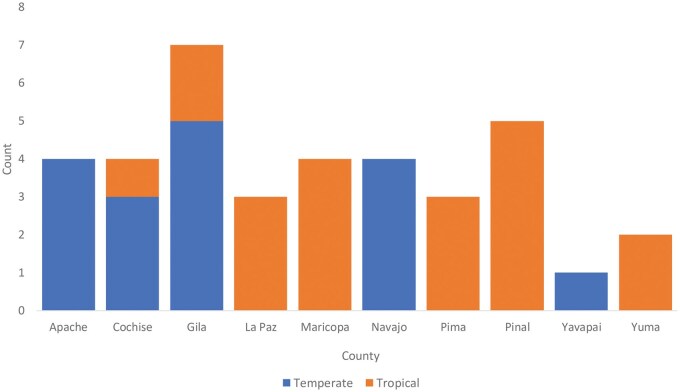
County of collection for *R. sanguineus* s.l. included in high-throughput sequencing using *Coxiella* blocking primer.

Thirty-nine *R. sanguineus* s.l. ticks were sequenced (Table 1). The primer designed to block *Coxiella* was largely successful, reducing Coxiella reads to a median of 0.2% and mean of 13% of reads per tick, compared to a mean of 48% in our a previous work conducted without the blocking primer (Brophy, Walker et al., 2022).Temperate lineage ticks tended to have more *Coxiella* after use of the blocking primer, suggesting either that the primer was less efficient in blocking that strain of the bacteria, or that temperate lineages may host a larger amount of *Coxiella*. After excluding the remaining *Coxiella* from the dataset and rarefying, 2 samples were removed due to low read counts. The final dataset included 37 ticks; 17 (46%) temperate lineage and 20 (54%) tropical lineage *R. sanguineus* s.l. ticks from ten Arizona counties (Fig. 1). With the exception of 2 nymphal samples, all were adults. There was a nearly even split between sexes—18 (51.4%) were female, and 17 (48.6%) were male. Read depths ranged from 4,068 to 104,492 reads per sample. After rarefying the data to 11,197 reads, there were 2,300 SVs and 640 bacterial genera detected among the 37 ticks included in the analysis.

**Table 1.** Life stage & sex of *R. sanguineus* s.l. included in high-throughput sequencing using *Coxiella* blocking primer.

Life stage	*N* (%)
**Nymph**	2 (5.4)
**Adult**	35 (94.6)
** Male**	17 (55.8)
** Female**	18 (44.2)
** Unfed**	10 (55.6)
** Partial**	7 (38.9)
** Fully**	1 (5.6)

**Fig. 2.**
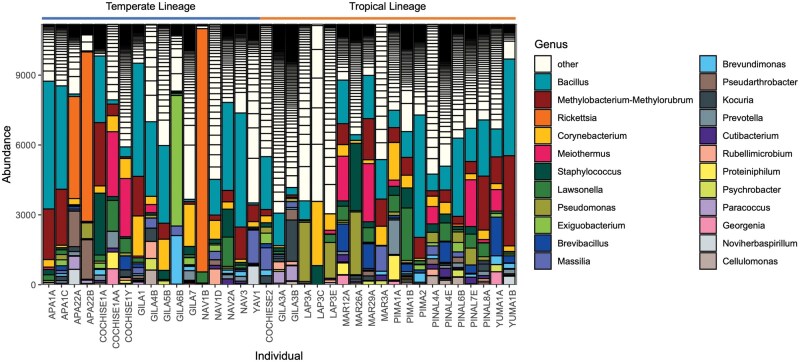
Abundances of bacterial genera in *R. sanguineus* s.l. ticks. Abundance of top 23 bacterial genera present in R. sanguineus s.l. ticks from Arizona. Each bar represents sequences derived from a tick after rarefaction to 11,197 reads, with genera indicated by colors. Genera that did not reach a relative abundance of 2.5% within at least 3 samples are labelled “Other.”

**Fig. 3.**
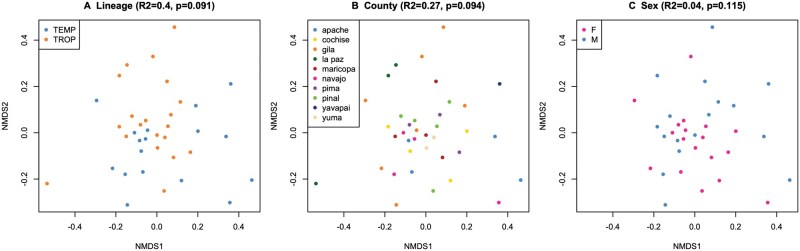
Ordinations of bacterial community composition using nonmetric multi-dimensional scaling (NDMS) plots of the Bray-Curtis dissimilarities between A) lineage, B) county of collection, and C) sex. Bacterial community structure is not significantly associated with any of these factors. Two nymphs are not displayed in panel C because they could not be sexed.

**Fig. 4.**
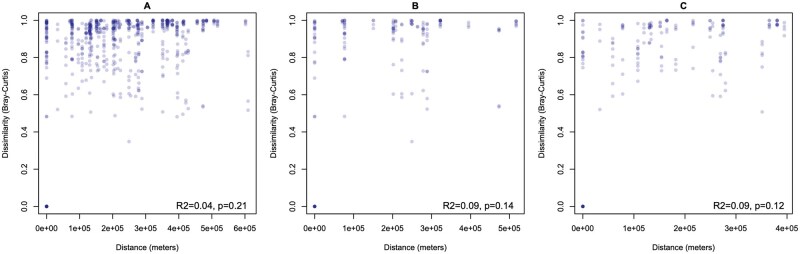
Plot of the Bray–Curtis distance between *R. sanguineus* s.l. ticks as a function of geographical distance. Bray–Curtis dissimilarity distance as a function of geographical distance calculated between coordinates for each sample based on the nearest city or town to the collection location. A) All samples, B) temperate lineage, C) tropical lineage. R-squared and *P* values are from Mantel tests.

The top 10 bacterial genera present were: *Bacillus, Methylobacterium-Methylorubrum, Rickettsia, Corynebacterium, Meiothermus, Staphylococcus, Lawsonella, Pseudomonas*, *Exiguobacterium*, and an unnamed genus in the Bacillaceae. (Table 2, Fig. 2). These genera accounted for 49% of all reads. Each remaining genus accounted for ≤2% of reads. Bacterial community composition did not differ according to *R. sanguineus* s.l. lineages, sex, or female engorgement levels (PERMANOVA; statistics shown in Table 3, Fig. 3). While overall beta diversity was not significantly associated with tick lineage, temperate and tropical ticks differed in the relative abundance of 30 bacterial genera (DeSeq2; Supplementary Fig. S1). These genera were: *Rickettsia, Deinococcus Ralstonia Flaviaesturariibacter, Craurococcus-Caldovatus, Pelomonas, Galbitalea, Enterococcus*, TM7a*, Enterobacter, Flavisolibacter, ­Sporosarcina, Rhodoferax, Brachybacterium, Anaerobacillus, Porphyromonas, Rubrobacter, Burkholderia-Caballeronia-Paraburkholderia, Actinoplanes, Enteractinococcus*, UCG-005, and nine other genera that were unnamed in the Silva database. Contrary to our hypothesis, there was no significant association between geographic distance and bacterial ­community dissimilarity (Mantel test, *r* = 0.0082, *P* = 0.387, Fig. 4). When calculated within lineage, there were still no significant differences in community structure by distance (*r* = 0.0623, *P* = 0.184 for temperate lineage, *r* = 0.0563, *P* = 0.25 for tropical lineage).

**Table 2.** Top 10 most abundant bacterial genera among *R. sanguineus* s.l. ticks.

Class	Order	Family	Genus	Total Reads	Number of Samples (%)	Percent of Reads
***Bacilli***	*Bacillales*	*Bacillaceae*	*Bacillus*	67,240	34	92%
***Actinobacteria***	*Corynebacteriales*	*Corynebacteriaceae*	*Corynebacterium*	19,165	33	89%
***Bacilli***	*Staphylococcales*	*Staphylococcaceae*	*Staphylococcus*	12,733	32	86%
***Alphaproteobacteria***	*Rhizobiales*	*Beijerinckiaceae*	*Methylobacterium-Methylorubrum*	28,675	28	76%
***Actinobacteria***	*Corynebacteriales*	*Corynebacteriaceae*	*Lawsonella*	12,182	28	76%
***Bacilli***	*Bacillales*	*Bacillaceae*	*NA*	7,444	28	76%
***Gammaproteobacteria***	*Pseudomonadales*	*Pseudomonadaceae*	*Pseudomonas*	10,937	27	73%
***Alphaproteobacteria***	*Sphingomonadales*	*Sphingomonadaceae*	*Sphingomonas*	5,837	26	70%
***Actinobacteria***	*Propionibacteriales*	*Propionibacteriaceae*	*Cutibacterium*	2,924	24	65%
***Gammaproteobacteria***	*Pseudomonadales*	*Moraxellaceae*	*Psychrobacter*	2,345	24	65%

Numbers are reported after rarefaction to 5,000 reads per sample. Top genera are ordered by number of reads

**Table 3.** PERMANOVA results based on Bray–Curtis dissimilarities in bacterial community structure by *R. sanguineus s.l*. lineage, sex, engorgement, and county.

Variable	D.F.	Sum of squares	*F*	*R* ^2^	*P* value
**Lineage**	**1**	**0.423**	**1.170**	**0.031**	**0.198**
**Residuals**	37	13.376			
**Sex**	**2**	**0.857**	**1.192**	**0.062**	**0.144**
**Residuals**	36	12.942		0.938	
**Engorgement**	**1**	**1.060**	**0.958**	**0.161**	**0.549**
**Residuals**	18	5.533		0.839	
**County**	**9**	**3.480**	**1.087**	**0.252**	**0.167**
**Residuals**	29	10.319		0.748	
***Multivariate analysis***					
**Lineage**	1	0.423	1.195	0.031	0.165
**County**	9	3.472	1.090	0.252	0.175

Bacterial community structure is not significantly associated with any of these factors.

Seven *Rickettsia* SVs were present within the dataset. A single *Rickettsia* SV was found at high abundance in 3 samples, all temperate lineage ticks. An NCBI Nucleotide BLAST query could not distinguish the sequence on a level finer than genus because many members of the *Rickettsia* have identical sequence in the amplicon region (Johnson et al. 2008). The other *Rickettsia* SVs were present at low abundance in single samples. Since only 3 samples had high abundance *Rickettsia*, it is not possible to make any conclusions about the bacterium’s effect on bacterial community structure.

## Discussion

This research compared the bacterial community structure of temperate and tropical *R. sanguineus* s.l. ticks in Arizona. The utilization of a blocking primer to inhibit the amplification of the dominant CLEs allowed for better insight into the rich bacterial microbiome of *R. sanguineus* s.l. ticks. We found many commonalities in bacterial genera among *R. sanguineus* s.l. ticks in Arizona. The majority of samples had *Bacillus, Methylobacterium-Methylorubrum, Rickettsia, Corynebacterium, Meiothermus, Staphylococcus, Lawsonella, Pseudomonas*, and *Exiguobacterium.* In spite of a very diverse community in which approximately 95% of bacterial genera each represent <1% of total reads, there were no clear distinctions in bacterial community composition based on lineage, sex, female engorgement level, or county of collection.

Our findings are in line with other research, which suggests that when the primary symbiont is excluded from analysis, the tick microbiome is highly variable (Lalzar et al. 2012, Ross et al. 2018, Binetruy et al. 2020). Some research on the *R. sanguineus* s.l. microbiome has indicated trends by lineage; however, such findings may be confounded by sampling the two lineages in distinct geographic areas (René-Martellet et al. 2017, de Cassia Luzzi et al. 2021). While some bacterial genera in our study differed in abundance between temperate and tropical ticks, these results must be interpreted with caution, as high abundance of a given bacterium in just a few samples could result in significant statistical findings.

Despite the near ubiquity of a few bacterial genera (*Bacillus* and *Methylobacterium-Methylorubrum*) across our samples, it appears likely that many members of *R. sanguineus* s.l.’s microbiome are transient and highly variable. Arthropods that have obligate, vertically transmitted symbionts often lack other stably-associated microbiota (Hammer et al. 2017, 2019, Ross et al. 2018). Many of the most abundant genera identified in our ticks are generalist bacteria that are often found in the environmental space that ticks occupy. Most tick species rely on a single maternally inherited endosymbiont for synthesis of B vitamins and cofactors lacking in their diet (Gottlieb et al. 2015, Smith et al. 2015, Ramaiah et al. 2017, Ben-yosef et al. 2020, Duron and Gottlieb 2020). In the presence of such a vital specialist, such as *R. sanguineus* s.l.*’*s *Coxiella*-like endosymbiont, it is unsurprising that the other members of the tick’s microbiome are generalist bacteria and may not play as important roles in host fitness.

Parsing out which bacteria may be hitchhikers from the ticks’ local environment or their host dogs and which are true representatives of the microbiome would be no easy task. Off-host ticks spend a significant amount of their time at ground level, for example, and may therefore acquire soil-associated bacteria (Zimmer et al. 2013, Narasimhan et al. 2021, Rojas-Jaimes et al. 2021). The genera *Staphylococcus* and *Cutibacterium* are sometimes removed from high-throughput sequencing analysis because of their close association with human skin, and we chose to remove two universally present SVs belonging to these genera from our data as contaminants. However, these are also common members of the canine skin and ear microbiomes (Tang et al. 2020). The ticks could plausibly have acquired these bacteria during feeding, which would be consistent with their prevalence in our samples.

This study adds insight to the presently sparse literature regarding the bacterial community composition of *R. sanguineus* s.l. We found that generalist and environment-associated bacteria accounted for the majority of the ticks’ bacterial community, with no systematic patterns with geography or between the temperate and tropical tick lineages. The results presented here ­suggest that ticks within a comparatively small region such as Arizona tend to acquire similar environmental bacteria. It is therefore unlikely that these secondary members of the *R. sanguineus* s.l. microbiome meaningfully influence vector competence of either of the 2 lineages in Arizona, particularly for *Rickettsia rickettsii* (the causal agent of Rocky Mountain Spotted Fever), for which they are a local vector. Future research on should focus on *R. sanguineus* s.l.’s obligate, maternally inherited symbionts, CLEs, and whether they may impact the tick’s ability to acquire and transmit pathogens such as *R. rickettsii.*

## Supplementary Material

tjaf186_Supplementary_Data

## Data Availability

Raw Illumina sequences will be published to the NCBI Sequence Read Archive.

## References

[tjaf186-B1] Abraham NM , Liu L, Lyon B, et al. 2017. Pathogen-mediated manipulation of arthropod microbiota to promote infection. PNAS 10:E781–E790. 10.1073/pnas.1613422114 PMC5293115 28096373

[tjaf186-B2] Agwunobi DO , Kamani J, Zheng H, et al. 2021. Bacterial diversity in *Rhipicephalus sanguineus* (Acari : Ixodidae) from two states in Nigeria. J. Entolog. Sci. 56:256–271.

[tjaf186-B3] Anders S , Huber W. 2010. Differential expression analysis for sequence count data. Nat. Prec. 10.1186/gb-2010-11-10-r106 PMC3218662 20979621

[tjaf186-B4] Andreotti R , De León AAP, Dowd SE, et al. 2011. Assessment of bacterial diversity in the cattle tick Rhipicephalus (Boophilus) microplus through tag-encoded pyrosequencing. *BMC Microbiol*. 11:6. 10.1186/1471-2180-11-6 PMC3025832 21211038

[tjaf186-B5] Ben-yosef M , Rot A, Mahagna M, et al. 2020. Coxiella -like endosymbiont of *Rhipicephalus sanguineus* is required for physiological processes during ontogeny. Front. Microbiol. 11:493–416. 32390951 10.3389/fmicb.2020.00493 PMC7188774

[tjaf186-B6] Binetruy F , Buysse M, Lejarre Q, et al. 2020. Microbial community structure reveals instability of nutritional symbiosis during the evolutionary radiation of Amblyomma ticks. Mol. Ecol. 29:1016–1029. 32034827 10.1111/mec.15373

[tjaf186-B7] Bonnet SI , Binetruy F, Hernández-Jarguín AM, et al. 2017. The tick microbiome: why non-pathogenic microorganisms matter in tick biology and pathogen transmission. Front. Cell. Infect. Microbiol. 7:236–214. 28642842 10.3389/fcimb.2017.00236 PMC5462901

[tjaf186-B8] Bonnet SI , Pollet T. 2020. Update on the intricate tango between tick microbiomes and tick-borne pathogens. Parasite Immunol. 43:1–12. 10.1111/pim.12813 33314216

[tjaf186-B9] Brophy M , Riehle MA, Mastrud N, et al. 2022. Genetic variation in *Rhipicephalus sanguineus* s. l. ticks across Arizona. Int. J. Environ. Res. Public Health 19:1–12. 10.3390/ijerph19074223 PMC8998742 35409903

[tjaf186-B10] Brophy M , Walker KR, Adamson JE, et al. 2022. Tropical and temperate lineages of *Rhipicephalus sanguineus* s. l. ticks (Acari : Ixodidae) host different strains of Coxiella-like endosymbionts. J. Med. Entomol. 59:2022–2029. 36124671 10.1093/jme/tjac132

[tjaf186-B11] Brownlie JC , Johnson KN. 2009. Symbiont-mediated protection in insect hosts. Trends Microbiol. 17:348–354. 19660955 10.1016/j.tim.2009.05.005

[tjaf186-B12] Budachetri K , Browning RE, Adamson SW, et al. 2014. An insight into the microbiome of the *Amblyomma maculatum* (Acari: Ixodidae). J. Med. Entomol. 51:119–129. 24605461 10.1603/me12223 PMC3956751

[tjaf186-B13] Callahan BJ , McMurdie PJ, Rosen MJ, et al. 2016. DADA2: high-resolution sample inference from Illumina amplicon data. Nat. Methods. 13:581–583. 27214047 10.1038/nmeth.3869 PMC4927377

[tjaf186-B14] Carpi G , Cagnacci F, Wittekindt NE, et al. 2011. Metagenomic profile of the bacterial communities associated with *Ixodes ricinus* ticks. PLoS One. 6:e25604. 22022422 10.1371/journal.pone.0025604 PMC3192763

[tjaf186-B15] de Cassia Luzzi MDC , Amoroso L, De Carvalho L, et al. 2021. Analysis on the prokaryotic microbiome in females and embryonic cell cultures of *Rhipicephalus sanguineus* tropical and temperate lineages from two specific localities in Brazil. Brazilian J. Vet. Parasitol 30:1–15. 10.1590/S1984-29612021066 34378769

[tjaf186-B16] Chicana B , Couper LI, Kwan JY, et al. 2019. Comparative microbiome profiles of sympatric tick species from the far-Western United States. Insect Mol. Biol. 10:1–12. 10.3390/insects10100353 PMC6836157 31635285

[tjaf186-B17] Dantas-Torres F , Latrofa M, Annoscia G, et al. 2013. Morphological and genetic diversity of *Rhipicephalus sanguineus* sensu lato from the new and old worlds. Parasit. Vectors. 6:213. 23880226 10.1186/1756-3305-6-213 PMC3735430

[tjaf186-B18] Dantas-Torres F , Otranto D. 2015. Further thoughts on the taxonomy and vector role of Rhipicephalus sanguineus group ticks. Vet. Parasitol. 208:9–13. 25579394 10.1016/j.vetpar.2014.12.014

[tjaf186-B19] Davis NM , Proctor DM, Holmes SP, et al. 2018. Simple statistical identification and removal of contaminant sequences in marker-gene and metagenomics data. Microbiome 6:226. 30558668 10.1186/s40168-018-0605-2 PMC6298009

[tjaf186-B20] Duron O , Binetruy F, Noël V, et al. 2017. Evolutionary changes in symbiont community structure in ticks. Mol. Ecol. 26:2905–2921. 28281305 10.1111/mec.14094

[tjaf186-B21] Duron O , Gottlieb Y. 2020. Convergence of nutritional symbioses in obligate blood feeders. Trends Parasitol. 36:816–825. 32811753 10.1016/j.pt.2020.07.007

[tjaf186-B22] Duron O , Noël V, McCoy KD, et al. 2015. The Recent evolution of a maternally-inherited endosymbiont of ticks led to the emergence of the Q fever pathogen, *Coxiella burnetii*. PLoS Pathog. 11:e1004892. 25978383 10.1371/journal.ppat.1004892 PMC4433120

[tjaf186-B23] Gall CA , Reif KE, Scoles GA, et al. 2016. The bacterial microbiome of *Dermacentor andersoni* ticks influences pathogen susceptibility. ISME J. 10:1846–1855. 26882265 10.1038/ismej.2015.266 PMC5029153

[tjaf186-B24] Gofton AW , Oskam CL, Lo N, et al. 2015. Inhibition of the endosymbiont “Candidatus Midichloria mitochondrii” during 16S rRNA gene profiling reveals potential pathogens in Ixodes ticks from Australia. Parasit. Vectors 8:1–11. 26108374 10.1186/s13071-015-0958-3 PMC4493822

[tjaf186-B25] Gottlieb Y , Lalzar I, Klasson L. 2015. Distinctive genome reduction rates revealed by genomic analyses of two Coxiella-like endosymbionts in ticks. Genome Biol. Evol. 7:1779–1796. 26025560 10.1093/gbe/evv108 PMC4494066

[tjaf186-B26] Gray J , Dantas-Torres F, Estrada-Pena A, et al. 2013. Systematics and ecology of the brown dog tick, *Rhipicephalus sanguineus*. Ticks Tick. Borne Dis. 4:171–180. 23415851 10.1016/j.ttbdis.2012.12.003

[tjaf186-B27] Guizzo MG , Parizi LF, Nunes RD, et al. 2017. A Coxiella mutualist symbiont is essential to the development of *Rhipicephalus microplus*. Sci. Rep. 7:17554–17510. 29242567 10.1038/s41598-017-17309-x PMC5730597

[tjaf186-B28] Hamady M , Walker JJ, Harris JK, et al. 2008. Error-correcting barcoded primers for pyrosequencing hundreds of samples in multiplex. Nat. Methods. 5:235–237. 18264105 10.1038/nmeth.1184 PMC3439997

[tjaf186-B29] Hammer TJ , Janzen DH, Hallwachs W, et al. 2017. Caterpillars lack a resident gut microbiome. Proc. Natl. Acad. Sci. U S A 114:9641–9646. 28830993 10.1073/pnas.1707186114 PMC5594680

[tjaf186-B30] Hammer TJ , Sanders JG, Fierer N. 2019. Not all animals need a microbiome. FEMS Microbiol. Lett 366:1–11. 10.1093/femsle/fnz117 31132110

[tjaf186-B31] Hawlena H , Rynkiewicz E, Toh E, et al. 2013. The arthropod, but not the vertebrate host or its environment, dictates bacterial community composition of fleas and ticks. Isme J. 7:221–223. 22739493 10.1038/ismej.2012.71 PMC3526175

[tjaf186-B32] Johnson M , Zaretskaya I, Raytselis Y, et al. 2008. NCBI BLAST: a better web interface. Nucleic Acids Res. 36:W5–W9. 18440982 10.1093/nar/gkn201 PMC2447716

[tjaf186-B33] Klindworth A , Pruesse E, Schweer T, et al. 2013. Evaluation of general 16S ribosomal RNA gene PCR primers for classical and next-generation sequencing-based diversity studies. Nucleic Acids Res. 41:e1. 22933715 10.1093/nar/gks808 PMC3592464

[tjaf186-B34] de la Fuente J , Antunes S, Bonnet S, et al. 2017. Tick-pathogen interactions and vector competence: identification of molecular drivers for tick-borne diseases. Front. Cell. Infect. Microbiol. 7:114–113. 28439499 10.3389/fcimb.2017.00114 PMC5383669

[tjaf186-B35] de la Fuente J , Blouin EF, Kocan KM. 2003. Infection exclusion of the rickettsial pathogen *Anaplasma marginale* in the tick vector *Dermacentor variabilis*. Clin. Diagn. Lab. Immunol. 10:182–184. 12522060 10.1128/CDLI.10.1.182-184.2003 PMC145288

[tjaf186-B36] Lalzar I , Harrus S, Mumcuoglu KY, et al. 2012. Composition and seasonal variation of *Rhipicephalus turanicus* and *Rhipicephalus sanguineus* bacterial communities. Appl. Environ. Microbiol. 78:4110–4116. 22467507 10.1128/AEM.00323-12 PMC3370557

[tjaf186-B37] Macaluso KR , Sonenshine DE, Ceraul SM, et al. 2002. Rickettsial infection in *Dermacentor variabilis* (Acari: Ixodidae) inhibits transovarial transmission of a second Rickettsia. J. Med. Entomol. 39:809–813. 12495176 10.1603/0022-2585-39.6.809

[tjaf186-B38] Martin M. 2011. Cutadapt removes adapter sequences from high-throughput sequencing reads. EMBnet. J. 17:10–12.

[tjaf186-B39] McMurdie PJ , Holmes S. 2013. Phyloseq: an R package for reproducible interactive analysis and graphics of microbiome census data. PLoS One. 8:e61217. 23630581 10.1371/journal.pone.0061217 PMC3632530

[tjaf186-B40] Moran NA , McCutcheon JP, Nakabachi A. 2008. Genomics and evolution of heritable bacterial symbionts. Annu. Rev. Genet. 42:165–190. 18983256 10.1146/annurev.genet.41.110306.130119

[tjaf186-B41] Mushegian A , Ebert D. 2016. Prospects & overviews rethinking ‘“mutualism”’ in diverse host-symbiont communities. Bioessays. 38:100–108. 26568407 10.1002/bies.201500074

[tjaf186-B42] Narasimhan S , Fikrig E. 2015. Tick microbiome: the force within. Trends Parasitol. 31:315–323. 25936226 10.1016/j.pt.2015.03.010 PMC4492851

[tjaf186-B43] Narasimhan S , Rajeevan N, Liu L, et al. 2014. Article gut microbiota of the tick vector *Ixodes scapularis* modulate colonization of the lyme disease spirochete. Cell Host Microbe. 15:58–71. 24439898 10.1016/j.chom.2013.12.001 PMC3905459

[tjaf186-B44] Narasimhan S , Swei A, Abouneameh S, et al. 2021. Grappling with the tick microbiome. Trends Parasitol. 37:722–733. 33962878 10.1016/j.pt.2021.04.004 PMC8282638

[tjaf186-B45] Nava S , Beati L, Venzal JM, et al. 2018. *Rhipicephalus sanguineus* (Latreille, 1806): neotype designation, morphological re-description of all parasitic stages and molecular characterization. Ticks Tick. Borne Dis. 9:1573–1585. 30100385 10.1016/j.ttbdis.2018.08.001

[tjaf186-B46] Oksanen J , Blanchet FG, Friendly M, et al. 2017. Vegan: community ecology package. R Package Version 2:4–2.

[tjaf186-B47] van Overbeek L , Gassner F, Van Der Plas CL, et al. 2008. Diversity of *Ixodes ricinus* tick-associated bacterial communities from different forests. FEMS Microbiol. Ecol. 66:72–84. 18355299 10.1111/j.1574-6941.2008.00468.x

[tjaf186-B48] Ramaiah A , Williams-Newkirk AJ, Frace MA, et al. 2017. Evolution of the genomes of the Coxiella-like endosymbionts of ticks. J. Mississippi Acad. Sci 62:21.

[tjaf186-B49] René-Martellet M , Minard G, Massot R, et al. 2017. Bacterial microbiota associated with *Rhipicephalus sanguineus* (s.l.) ticks from France, Senegal and Arizona. Parasit. Vectors. 10:416. 10. 28886749 10.1186/s13071-017-2352-9 PMC5591579

[tjaf186-B50] Rohland N , Reich D. 2012. Cost-effective, high-throughput DNA sequencing. Genome Res. 22:939–946. 22267522 10.1101/gr.128124.111 PMC3337438

[tjaf186-B51] Rojas-Jaimes J , Lindo-Seminario D, Correa-Nunes G, et al. 2021. Characterization of the bacterial microbiome of *Rhipicephalus* (Boophilus) microplus collected from Pecari tajacu “Sajino”. Madre. Sci. Rep 11:1–8. 10.1038/s41598-021-86177-3 PMC7988070 33758359

[tjaf186-B52] Ross BD , Hayes B, Radey MC, et al. 2018. *Ixodes scapularis* does not harbor a stable midgut microbiome. ISME J. 12:2596–2607. 29946195 10.1038/s41396-018-0161-6 PMC6194123

[tjaf186-B53] Rynkiewicz EC , Hemmerich C, Rusch DB, et al. 2015. Concordance of bacterial communities of two tick species and blood of their shared rodent host. Mol. Ecol. 24:2566–2579. 25847197 10.1111/mec.13187

[tjaf186-B54] Šlapeta J , Chandra S, Halliday B. 2021. The “Tropical Lineage” of the Brown Dog Tick Rhipicephalus sanguineus Sensu Lato Identified as Rhipicephalus linnaei (Audouin, 1826) q 51:431–436. 10.1016/j.ijpara.2021.02.001 33713653

[tjaf186-B55] Smith TA , Driscoll T, Gillespie JJ, et al. 2015. A Coxiella-like endosymbiont is a potential vitamin source for the lone star tick. Genome Biol. Evol. 7:831–838. 25618142 10.1093/gbe/evv016 PMC4994718

[tjaf186-B56] Steiner FE , Pinger RR, Vann CN, et al. 2008. Infection and co-infection rates of *Anaplasma phagocytophilum* variants, *Babesia* spp., *Borrelia burgdorferi*, and the rickettsial endosymbiont in *Ixodes scapularis* (Acari : Ixodidae) from sites in Indiana, Maine, Pennsylvania, and Wisconsin. J. me 45:289–297. 10.1603/0022-2585(2008)45[289:iacroa]2.0.co;2 18402145

[tjaf186-B57] Tang S , Prem A, Tjokrosurjo J, et al. 2020. The canine skin and ear microbiome : a comprehensive survey of pathogens implicated in canine skin and ear infections using a novel next-generation- sequencing-based assay. Vet. Microbiol. 247:108764. 32768216 10.1016/j.vetmic.2020.108764

[tjaf186-B58] R Team. 2014. R: a language and environment for statistical computing. R Found. Stat. Comput. http://www.r-project.org/.

[tjaf186-B59] Thapa S , Zhang Y, Allen MS. 2019. Bacterial microbiomes of *Ixodes scapularis* ticks collected from Massachusetts and Texas, USA. BMC Microbiol. 19:138–112. 31234774 10.1186/s12866-019-1514-7 PMC6591839

[tjaf186-B60] Vestheim H , Deagle BE, Jarman SN. 2011. Application of blocking oligonucleotides to improve signal-to-noise ratio in a PCR. Methods Mol. Biol. 687:265–274. 20967615 10.1007/978-1-60761-944-4_19

[tjaf186-B61] Walker JB , Keirans JE, Horak IG. 2000. The genus Rhipicephalus (Acari, Ixodidae). Cambridge University Press.

[tjaf186-B62] Wang Q , Garrity GM, Tiedje JM, et al. 2007. Naïve Bayesian classifier for rapid assignment of rRNA sequences into the new bacterial taxonomy. Appl. Environ. Microbiol. 73:5261–5267. 17586664 10.1128/AEM.00062-07 PMC1950982

[tjaf186-B63] Williams-Newkirk AJ , Rowe LA, Mixson-hayden TR, et al. 2014. Characterization of the bacterial communities of life stages of free living lone star ticks (*Amblyomma americanum*). PLoS One. 9:e102130. 25054227 10.1371/journal.pone.0102130 PMC4108322

[tjaf186-B64] Yilmaz P , Parfrey LW, Yarza P, et al. 2014. The SILVA and “all-species Living Tree Project (LTP)” taxonomic frameworks. Nucleic Acids Res. 42:D643–648. 24293649 10.1093/nar/gkt1209 PMC3965112

[tjaf186-B65] Zhang X , Yang Z, Lu B, et al. 2014. The composition and transmission of microbiome in hard tick, *Ixodes persulcatus*, during blood meal. Ticks Tick. Borne Dis. 5:864–870. 25150725 10.1016/j.ttbdis.2014.07.009

[tjaf186-B66] Zimmer KR , Seixas A, Conceicao JM, et al. 2013. Cattle tick-associated bacteria exert anti-biofilm and anti-*Tritrichomonas foetus* activities. Vet. Microbiol. 164:171–176. 23434012 10.1016/j.vetmic.2013.01.029

[tjaf186-B67] Zolnik CP , Prill RJ, Falco RC, et al. 2016. Microbiome changes through ontogeny of a tick pathogen vector. Mol. Ecol. 25:4963–4977. 27588381 10.1111/mec.13832

[tjaf186-B68] Zug R , Hammerstein P. 2015. Bad guys turned nice? A critical assessment of *Wolbachia mutualisms* in arthropod hosts. Biol. Rev. Camb. ­Philos. Soc. 90:89–111. 10.1111/brv.12098 24618033

