# Supplementary figures and images for: Rhipicephalus sanguineus s.l. ticks (Acari: Ixodidae) harbor non-divergent bacterial microbiomes in Arizona
Source: J Med Entomol. 2026 Jan 22;63(1):tjaf186. doi: 10.1093/jme/tjaf186 (PMC12828283; doi:10.1093/jme/tjaf186)

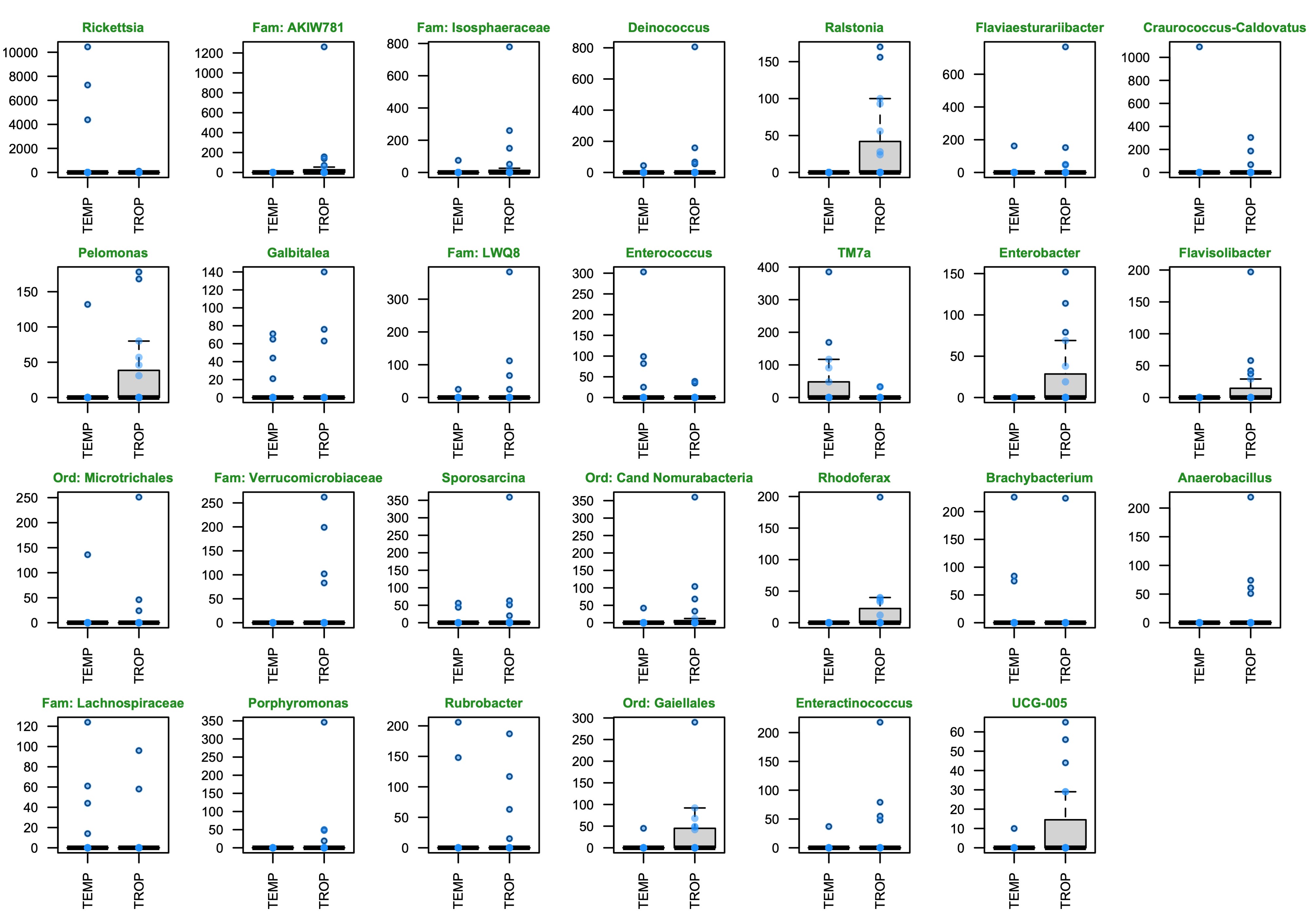

Supplement: tjaf186_Supplementary_Data [file tjaf186_supplementary_data.jpeg]
